# Isolation of Left Common Carotid Artery with Its Origin Proximal to Patent Ductus Arteriosus Presenting in Adult Age

**DOI:** 10.1155/2016/4149365

**Published:** 2016-04-26

**Authors:** Anagha R. Joshi, Saurabh Joshi, Kiran Kale, Rahul Jain, Jernail Singh Bava

**Affiliations:** Department of Radiology, Lokmanya Tilak Municipal Medical College and General Hospital, Sion West, Mumbai, Maharashtra 400022, India

## Abstract

Anomalies of aortic arch are a common occurrence. Such anomalies of right sided aortic arch with its various branching patterns are of clinical importance. Rarer anomalies include isolation (deficient connection) of either left subclavian artery or left common carotid artery; that is, they do not have their origin from aorta or its major branches. We present a case of an 18-year-old male who presented with gradual onset pulsatile swelling with bruit in neck on left side and was evaluated by CT brain and neck angiography. CT angiography revealed right sided aortic arch with aberrant left subclavian artery and isolated left common carotid artery. Very few cases of such an anomaly have been documented in the literature but none in an adult.

## 1. Introduction

Anomalies of aortic arch are a common occurrence. Few of such anomalies are right sided aortic arch with its various branching patterns, double aortic arch or left sided arch with aberrant right subclavian artery with or without formation of a vascular ring and variants in branching patterns of aortic arch. Edwards' hypothetical double aortic arch explains aortic arch abnormalities by selective regression of various parts of either arch [1].

Right sided aortic arch can have mirror branching which is associated with congenital cardiac defects in 98% of the cases. Another variant is right sided aortic arch with aberrant left subclavian artery. Rare anomalies include isolation of either left subclavian artery, left common carotid artery, or innominate artery; that is, they do not have their origin from aorta or its major branches [2]. Isolation of left common carotid artery is the rarest of the three. We report a case of an isolated left common carotid artery in an 18-year-old patient with right sided aortic arch and aberrant left subclavian artery.

## 2. Case Report

An 18-year-old previously asymptomatic male came with complaints of swelling in the left side of neck which was gradual in onset and slowly increasing in size and no other major complaints. Patient did not have any neurological symptoms at present or in the past and had normal development. On examination, the swelling was found to be pulsatile with audible bruit over it.

CT brain and neck angiography was advised in view of suspicion of a cervical AV malformation.

**Figure 1:**
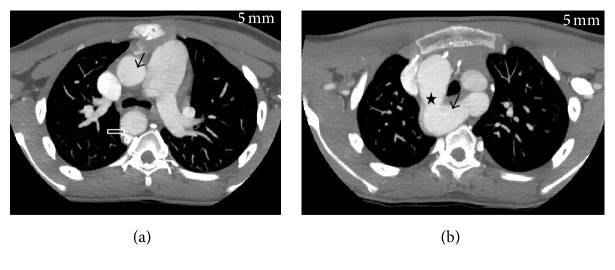
Axial MIP images at the level of ascending aorta and aortic arch showing right sided aortic arch and aberrant origin of left subclavian artery. (a) Ascending aorta (black arrow), descending aorta (open arrow), (b) aortic arch (star) and aberrant origin of left subclavian artery with diverticulum of Kommerell (black arrow).

**Figure 2:**
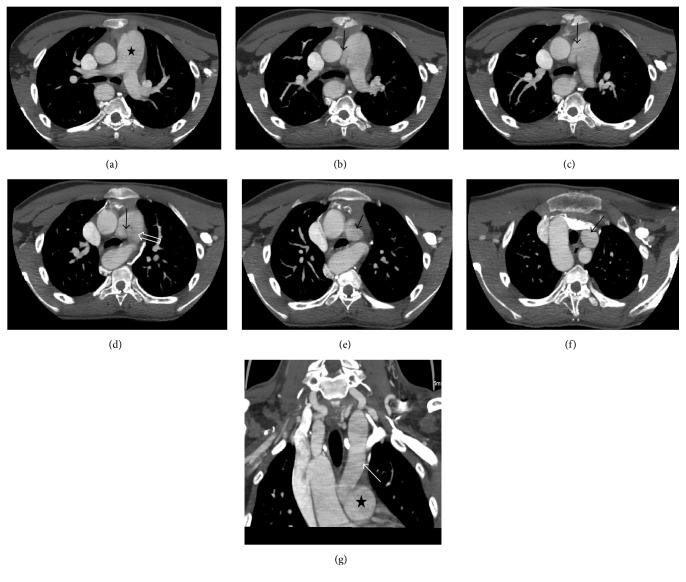
((a)–(f)) Sequential axial MIP images from the level of bifurcation of MPA (star) superiorly showing origin of left CCA from MPA (black arrow). (d) showing a portion of patent ductus arteriosus (open arrow). (g) Corresponding coronal reconstructed MIP image showing MPA (star) and origin of left CCA from MPA and its dilation (arrow).

CT angiography revealed right sided aortic arch (Figures 1(a) and 1(b)). Right common carotid artery and right subclavian artery were seen originating as the first and second branches of arch, respectively. Aberrant origin of left subclavian artery noted coursing posterior to oesophagus with mild dilatation was seen at the origin of left subclavian artery s/o diverticulum of Kommerell (Figure 1(b)). Left common carotid artery was not seen originating from arch of aorta. It was seen originating from right side of main pulmonary artery before its bifurcation (Figure 2).

A small connection was also seen between the main pulmonary artery and left common carotid artery apart from its origin. This could represent a portion of patent ductus arteriosus (Figure 2(d)).

Left common carotid artery showed moderate dilatation in the cervical region, measuring 3 cm before bifurcation.

**Figure 3:**
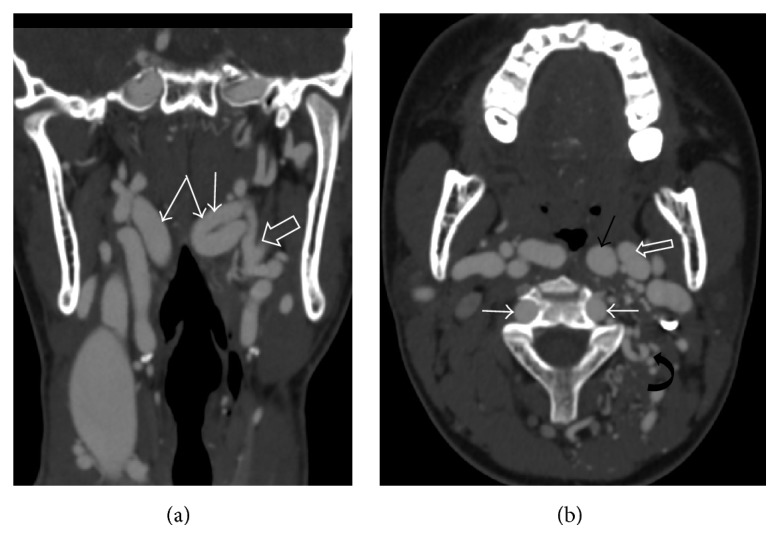
(a) Coronal MIP image showing dilatation and tortuosity of left ICA (single white arrow) and ECA (open arrow) and kissing carotids (white arrows). (b) Axial MIP image at the level of bifurcation of CCA showing dilated left ICA (black arrow), ECA (open arrow) with multiple inter and intramuscular arterial collaterals on left side of neck (curved arrow), and prominent vertebral arteries (white arrows).

Left internal carotid artery, left external carotid artery, and main branches of external carotid artery were found to be tortuous and dilated with multiple arterial inter- and intramuscular collaterals on left side of neck. Incidental finding noted was kissing carotids (bilateral internal carotid arteries) due to its medial course (Figures 3(a) and 3(b)).

Bilateral vertebral arteries were prominent in cervical region (Figure 3(b)).

There is no evidence of dilatation of main pulmonary artery or right atrium.

## 3. Discussion

Aortic arch anomalies have been classically explained by Edward's hypothetical double aortic arch and regression of its parts on right or left side leading to various combinations. Right sided aortic arch with aberrant left subclavian artery can be explained on the basis of regression in left arch involving dorsal segment between left common carotid artery and left subclavian arteries with regression of right ductus arteriosus. In such cases, left common carotid artery is seen as the first branch of the arch followed by right common carotid artery and right subclavian artery, respectively. However, as left common carotid artery was isolated in our case, right common carotid artery was seen as the first branch followed by right subclavian artery and aberrant left subclavian artery.

Only eight cases of isolated left common carotid artery have been documented in the literature [2–8] till now. None of these cases were reported in adults.

According to Männer et al. [2], isolation of any aortic arch artery is explained not only by the regression of arch proximal and distal segments to it, but also by “deficient regression” that is persistence of ductus arteriosus. Presence of ductus arteriosus explains the communication with the pulmonary artery whereas regression of both proximal and distal segments explains the noncommunication to the arch.

Though this explains the basic anomaly in our patient, there remains an issue about a small connection that is seen separate from the origin of left common carotid artery from pulmonary artery. Similar cases have been reported before by Fouilloux et al. and Kaushik et al. [4, 5]. According to Männer [3] and Kaushik et al. [5], these cannot be explained based only on the Edward's hypothetical double arch model. Both of these authors have proposed malseptation of truncoaortic sac as a possible mechanism, that is, a disturbance in the septation of the truncoaortic sac secondary to abnormal migration of neural crest cells. In our case, presence of the small connection can only be explained by the same malseptation concept.

Fouilloux et al. [4] proposed two alternative hypotheses to explain the embryology of their case. (a) The initial segment of the isolated LCCA may represent a patent left ductus arteriosus at an abnormal position, while the fibrous band may represent the atretic left aortic arch segment between the LCCA and the left subclavian artery. (b) The fibrous band may represent the remnant of the left ductus arteriosus.

Importance of the detection and description of this anomaly is twofold. This finding, although rare, is always associated with other structural heart defects and aberrant left subclavian artery. Thus, when contemplating surgical corrections, it will play a major role in decision making. Secondly, with low pressure pulmonary circulation, a component of left to right shunting also develops. This is seen in our case as multiple collateral channels in the territory of left common carotid artery.

Such a case has not been described in an adult before. Therefore, our case should serve as an indicator of progression of cases diagnosed in infancy and childhood.

## 4. Conclusion

Our case is unique in the sense that it is the first case reported in an adult along with possible presence of a small connection being explained only by malseptation of truncoaortic sac along with regression of segments and persistence of ductus arteriosus. Therefore, our case should serve as indicator of progression of cases diagnosed in infancy and childhood.
